# The QKI-6 and QKI-7 RNA Binding Proteins Block Proliferation and Promote Schwann Cell Myelination

**DOI:** 10.1371/journal.pone.0005867

**Published:** 2009-06-11

**Authors:** Daniel Larocque, Gabriela Fragoso, Jinghan Huang, Walter E. Mushynski, Martin Loignon, Stéphane Richard, Guillermina Almazan

**Affiliations:** 1 Terry Fox Molecular Oncology Group and the Bloomfield Center for Research on Aging, Lady Davis Institute for Medical Research, Sir Mortimer B. Davis Jewish General Hospital, Department of Oncology and Medicine, McGill University, Montréal, Québec, Canada; 2 Department of Pharmacology and Therapeutics, McGill University, Montréal, Québec, Canada; 3 Department of Biochemistry, McGill University, Montréal, Québec, Canada; Universidade Federal do Rio de Janeiro (UFRJ), Instituto de Biofísica da UFRJ, Brazil

## Abstract

**Background:**

The *quaking viable* (*qk^v^*) mice have uncompacted myelin in their central and peripheral nervous system (CNS, PNS). The *qk* gene encodes 3 major alternatively spliced isoforms that contain unique sequence at their C-terminus dictating their cellular localization. QKI-5 is a nuclear isoform, whereas QKI-6 and QKI-7 are cytoplasmic isoforms. The *qk^v^* mice harbor an enhancer/promoter deletion that prevents the expression of isoforms QKI-6 and QKI-7 in myelinating cells resulting in a dysmyelination phenotype. It was shown that QKI regulates the differentiation of oligodendrocytes, the myelinating cells of the CNS, however, little is known about the role of the QKI proteins, or RNA binding proteins in PNS myelination.

**Methodology/Principal Findings:**

To define the role of the QKI proteins in PNS myelination, we ectopically expressed QKI-6 and QKI-7 in primary rat Schwann cell/neuron from dorsal root ganglia cocultures. We show that the QKI isoforms blocked proliferation and promoted Schwann cell differentiation and myelination. In addition, these events were coordinated with elevated proteins levels of p27^KIP1^ and myelin basic protein (MBP), markers of Schwann cell differentiation. QKI-6 and QKI-7 expressing co-cultures contained myelinated fibers that had directionality and contained significantly thicker myelin, as assessed by electron microscopy. Moreover, QKI-deficient Schwann cells had reduced levels of MBP, p27^KIP1^ and Krox-20 mRNAs, as assessed by quantitative RT-PCR.

**Conclusions/Significance:**

Our findings suggest that the QKI-6 and QKI-7 RNA binding proteins are positive regulators of PNS myelination and show that the QKI RNA binding proteins play a key role in Schwann cell differentiation and myelination.

## Introduction

The *quaking (qk) viable* mice (*qk* ^v^) develop rapid tremors and clonic seizures resulting from their severe dysmyelination [1]. *Qk* ^v^ mice contain a deletion in the promoter and enhancer region of the *qk* gene that prevents proper expression of its encoding proteins [2]. Three major alternatively spliced transcripts are generated from the *qk* gene, encoding isoforms that differ in their C-terminal amino acid sequence. QKI-5 contains 30 unique amino acids that harbor a nuclear localization signal and thus QKI-5 is nuclear [3]. QKI-6 and QKI-7 contain 8 and 14 unique residues, respectively, and these isoforms are predominantly cytoplasmic [4]. The QKI isoforms homo- and heterodimerize [5] and their balanced expression in oligodendrocytes is required for proper myelination in the central nervous system (CNS) [6]. The QKI isoforms are also expressed in Schwann cells [7], the myelinating cells of the peripheral nervous system (PNS). The expression of QKI-5 is highest during embryogenesis and declines with age [2]. The QKI-6 and QKI-7 isoforms are expressed during late embryogenesis and their peak expression coincides with myelination at post-natal day 14 in mouse brain [2]. The *qk* promoter deletion observed in *qk^v^* mice mainly prevents the expression of QKI-6 and QKI-7 isoforms, hence the balance between the isoforms is lost and QKI-5 is the predominant isoform remaining [7]. It is thought that the lack of QKI-6 and QKI-7 expression is the reason for oligodendrocyte maturation defects and the dysmyelination, observed in *qk^v^* mice [7]. Consistent with this hypothesis, the ectopic expression of the QKI-6 and QKI-7 induces oligodendrocyte differentiation [8] and an oligodendrocyte-specific *QKI-6* transgenic allele rescues the CNS myelination defects of the *qk^v^* mice [9].

The QKI isoforms are members of the KH-type RNA binding protein family that bind specific RNA sequences with high affinity [10], [11], [12]. These QKI proteins can selectively interact with a short sequence termed the QKI response element (QRE) [13], [14], found in the 3′-untranslated region (3′-UTR) of the mRNAs encoding MBP [6], [15], Krox-20 (also called Egr-2) [13], [16] and MAP1B [17]. The sequence preference for QKI recognition is NACUAAY (with a space of N1-20) UAAY, where N is any nucleotide and Y is a pyrimidine [13]. Using the QKI RNA binding consensus sequence, a bioinformatics analysis led to the identification of binding sites in the p27^KIP1^ (cyclin-dependent kinase inhibitor), MBP and Krox-20 mRNAs and these sequences were bound by the QKI proteins *in vitro* with high affinity using gel-shift assays [13]. Moreover, the QKI isoforms associated with p27^KIP1^ mRNA *in vivo* and lead to the stabilization of the mRNA half-life in oligodendrocytes [8]. These findings provide evidence that the QKI proteins are regulators of post-transcriptional events during myelination.

Schwann cells in the PNS establish close contact with axons and produce myelin, a specialized membraneous structure that permits rapid nerve conduction [18], [19]. Neural crest-derived cells associate with the sensory axons growing out of the dorsal root ganglia (DRG) and with the CNS motor axons, and differentiate into Schwann cells precursor cells. These cells proliferate during their migration along the nerves and then escape from the cell cycle followed by an up-regulation of transcription factors and myelin proteins, forming compact myelin lamellae around the peripheral nerves [18], [19]. Several transcription factors are required for cell cycle arrest and differentiation of Schwann cells, including Krox-20 and Sox 10 [19]. The ability of Schwann cells to stop proliferating and induce the cell cycle inhibitor p27^KIP1^1 has been associated with Krox-20 [20]. The zinc finger transcription factor, Krox-20, is a master regulator essential for PNS myelination [21]. Krox-20 null mice or hypomorphic mutations block the myelination program at the premyelinating stage [21], [22]. The forced expression of Krox-20 results in cell cycle arrest and the activation of genes encoding structural myelin proteins and enzymes involved in lipid synthesis [23], [24]. Krox-20 is also required for myelin maintenance as its loss in adult mice results in severe demyelination and Schwann cell dedifferentiation [25]. Therefore, Schwann cell myelination is constantly controlled by both positive and negative factors [19], [26].

In the PNS of the *qk^v^* mouse, axons of sciatic nerves exhibit a separation of intraperiod lines in the myelin sheath, and a variation of Schmidt-Lanterman incisures [27], [28], [29]. Ventral root axons display a dilation of the periaxonal space of myelinated fibers and failure of Schwann cells to convert mesaxon membranes to compact myelin [30], [31]. These observations allow us to hypothesize that QKI proteins might be involved in Schwann cell myelination and differentiation. Despite the observations in the CNS, the ability of the QKI proteins to regulate PNS myelination is unknown. In this study we report that QKI-6 and QKI-7 induce Schwann cell maturation, by promoting the condensation of a thicker myelin. Moreover, QKI-deficient Schwann cells had reduced levels of MBP, p27^KIP1^ and Krox-20 mRNAs, as assessed by quantitative RT-PCR. These data demonstrate that the QKI-6 and QKI-7 proteins are potent regulators of PNS myelination.

## Results

### QKI-6 and QKI-7 promote cell cycle arrest of Schwann cells

**Figure 1:**
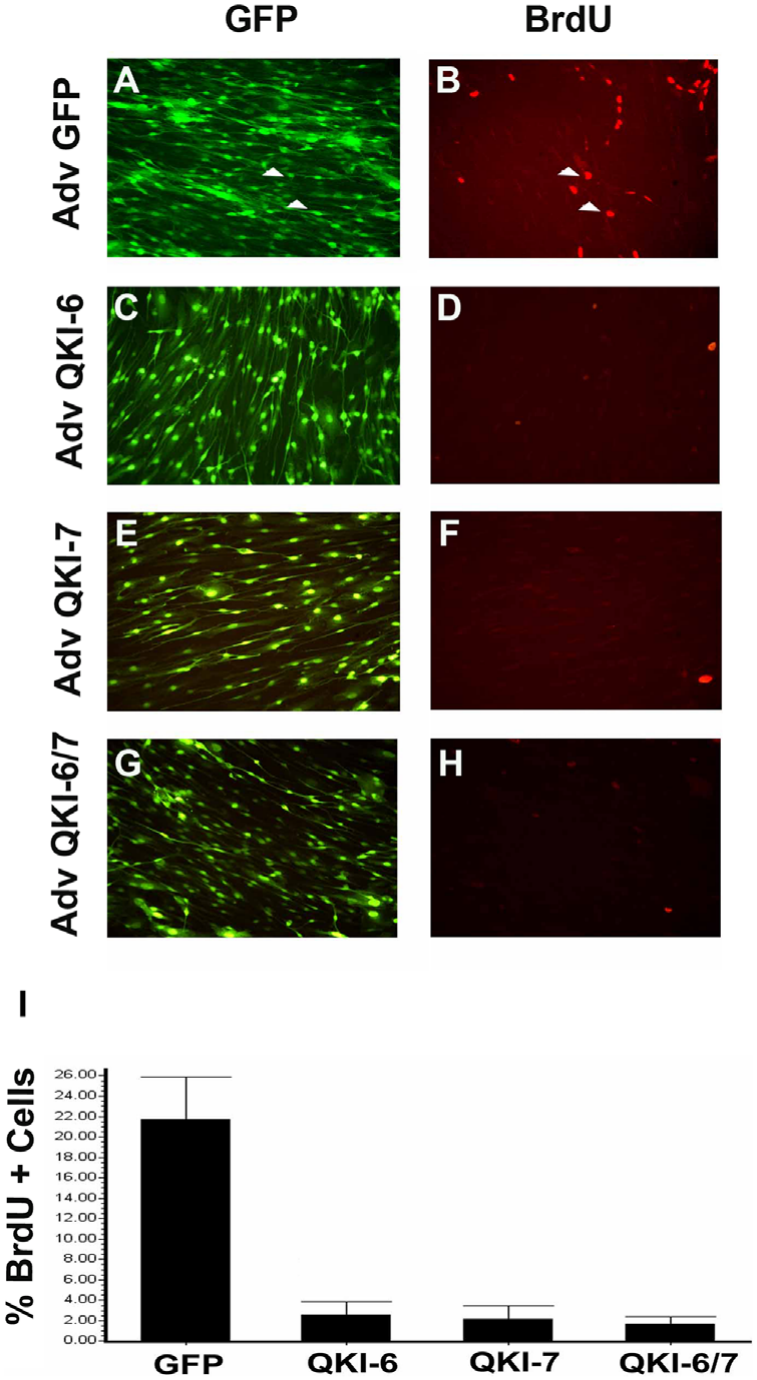
The QKI isoforms induce Schwann cell cycle arrest. Primary Schwann cells/neuron co-cultures were infected for 48 hr with control GFP adenovirus (A and B) and adenoviruses encoding for QKI-6 (C and D), QKI-7 (E and F), and the combination of QKI-6/7 (G and H). The green fluorescence denotes infected cells (panels A, C, E and G). Incorporation of BrdU (16 hr pulse) was detected with an anti-BrdU antibody followed by a goat anti-mouse conjugated to Alexa 546 (red) (B, D, F and H). Arrowheads denote the GFP positive Schwann cells that are also BrdU positive in the controls infected with GFP-adenovirus. (I). The expression of each QKI isoform causes inhibition of Schwann cell proliferation. The graph shows the percentage of GFP positive cells that are also BrdU positive. The proliferation was quantified from *n*>500 cells from three different experiments (*p*<0.001, ANOVA). The error bars represent +/− standard deviation of the mean.

The *quaking viable* mice (*qk^v^*) exhibit PNS dysmyelination [27], [28], [29]. Moreover, the expression of the cytoplasmic QKI-6 and QKI-7 is lost in the Schwann cells of *qk^v^* mice. These observations suggest that QKI-6 and QKI-7 may regulate Schwann cell differentiation [7]. To investigate the functional significance of QKI in Schwann cells, primary rat co-cultures of Schwann cells and neurons obtained from the dorsal root ganglia (DRG), which reflect the *in vivo* context of glial cells in the proximity of neurons [32], were transduced with adenoviruses expressing QKI-6 or QKI-7. Each virus also encodes the green fluorescent protein (GFP) for the microscopic monitoring of transduction efficiency and ∼95% of the Schwann cells were transduced, as visualized by the presence of the green fluorescence (data not shown and Figure 1). The adenovirus transduction resulted in an ∼6- to 8-fold increase in QKI-6 and QKI-7 protein expression (Figure S1). This elevated level of QKI-6 and QKI-7 expression is similar to what is observed in mice brains during the time of myelination in the CNS (Figure S2). In these conditions, the proliferation of Schwann cells was inhibited by either QKI-6 or QKI-7 or both QKI-6/7 isoforms, as determined by immunofluorescence detection of bromodeoxyuridine (BrdU) incorporation (Figure 1A–H). A quantitative analysis from three separate experiments to determine the percentage of BrdU-positive Schwann cells set the rate of incorporation at ∼3% and ∼2% for cells transduced with QKI-6 or QKI-7 expressing adenoviruses, respectively, and to ∼20% for cells transduced with the control vector GFP expressing adenovirus (Figure 1I). These findings demonstrate that both QKI-6 and QKI-7 isoforms inhibit Schwann cell proliferation.

### QKI-6 and QKI-7 induce Schwann cell maturation

**Figure 2:**
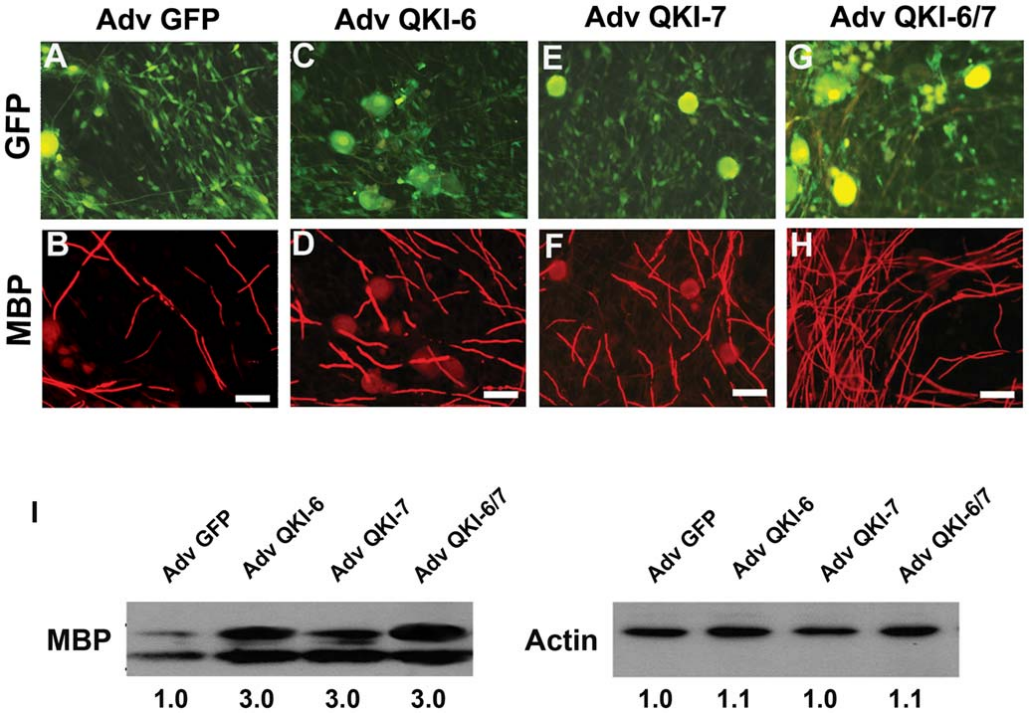
QKI-6 and QKI-7 induce the expression of Schwann cell differentiation markers and enhance their maturation. Co-cultures of Schwann cells/neurons were transduced with control adenoviruses (Adv GFP, A and B), adenoviruses encoding QKI-6 (C and D), QKI-7 (E and F), or a combination of QKI-6/7 (G and H). Myelination was induced for 5 days with ascorbic acid and the culture stained with anti-MBP antibodies (red). (I) Parallel cultures were lysed and analyzed by immunoblotting for MBP, and β-actin was used to ensure equal loading. The relative band intensities quantified by densitometry are indicated below.

As the QKI-6 and QKI-7 isoforms are known to promote oligodendrocyte maturation [8], we next examined whether these isoforms could regulate the differentiation of myelinating Schwann cells. The primary rat Schwann cell/DRGN co-cultures were transduced with control (GFP), QKI-6, QKI-7 and QKI-6/7 expressing adenoviruses. Myelination was initiated by the addition of ascorbic acid, a standard method of inducing myelination in these co-cultures [32]. Five days later, the co-cultures were examined for the expression of myelin basic proteins (MBP) by immunofluorescence and confocal microscopy. QKI-6 and QKI-7 led to a significant increase in the number of myelinated axonal segments, as compared to the GFP control cultures (Figure 2A–H). Compared to GFP-transduced co-cultures, the MBP protein expression was significantly increased (∼3-fold) in QKI-6, QKI-7 and QKI-6/7 transduced co-cultures, as observed by immunoblotting with anti-MBP antibodies and normalizing to β-actin levels (Figure 2I). These data demonstrate that QKI-6 and QKI-7 are positive inducers of Schwann cell differentiation.

### QKI-6 and QKI-7 are positive regulators of PNS myelination

**Figure 3:**
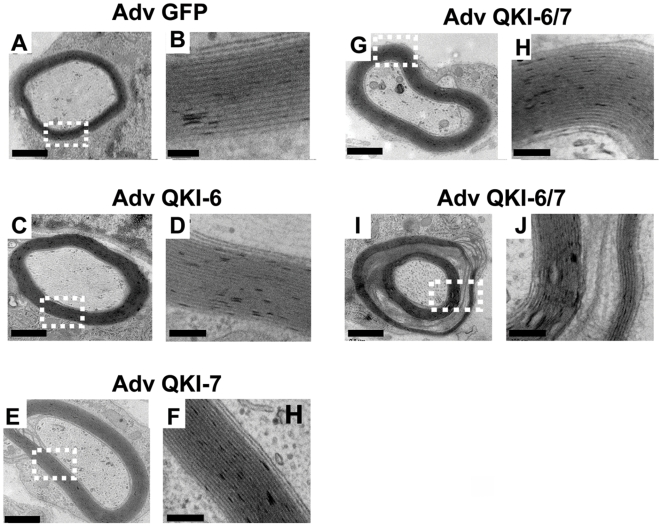
Electron microscopy of the myelin layers of the QKI-transduced Schwann cells/neurons co-cultures. Co-cultures of Schwann cells/neurons were transduced with adenoviruses encoding GFP alone (A and B), QKI-6 (C and D), QKI-7 (E and F), combination of QKI-6 and QKI-7 (G to J). Myelin layers were analyzed using electron microscopy. Each picture is a representative image from 30 different axons for each treatment showing a detailed view of the myelin sheath.

After the ascorbate treatment, the Schwann cells begin to elongate and to ensheath the neighboring axon. We measured the thickness of the myelin sheath on transverse sections of 5 day-old myelinated cultures, by electron microscopy (Figure 3). The myelin lamellae from cultures expressing QKI-6 (Figure 3C and D) or QKI-7 (Figure 3E and F) were dense, and the combination of QKI-6 and QKI-7 produced compact myelin with an increased number of wraps (Figure 3G and H). The control GFP infected cultures contained compact myelin with wraps separated by interperiodal lines and had a *g* ratio of 0.734±0.015 (Table 1). The expression of the individual QKI-6 and QKI-7 did not lead to a statistical significant increase in myelin (Table 1). On the other hand, the expression of both QKI-6 and QKI-7 led to a significant increase in myelin with a *g* ratio of 0.599±0.030. However, ∼21% of the myelinated internodes displayed various abnormalities (Figure 3I and J), including cytoplasm inclusion in the intraperiodal space. These ultrastructural data confirmed the positive regulatory role of QKI-6 and QKI-7 in PNS myelination.

**Table 1:** One way analysis of variance, *p*<0.0001 for *g* ratio.

Treatment	Number of measures	G ratios Mean±SEM
AdGFP	7	0.734±0.015
AdQKI-6	8	0.735±0.023
AdQKI-7	6	0.717±0.055
AdQKI-6/7	13	0.599±0.030 **

Dunnett multiple comparison test, AdGFP versus AdQKI-6/7.

** *p*<0.01.

Co-cultures of Schwann cells/neurons were transduced with adenovirus coding for different QKI isoforms. *G* ratio was determined from electron microscopy micrographs of cross-sections of 5-day old myelinated cultures.

### QKI-6 and QKI-7 induce p27^KIP1^ expression in myelinating Schwann cells

**Figure 4:**
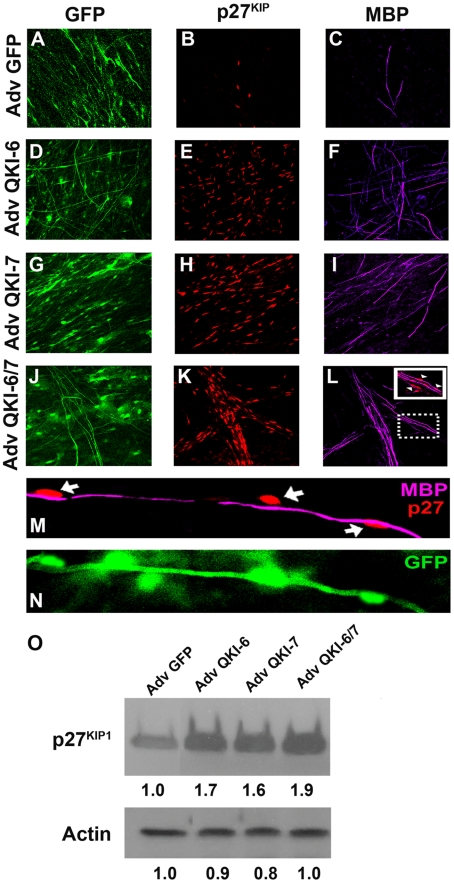
QKI-6/7 transduced Schwann cells/neurons co-cultures display elevated nuclear p27^KIP1^ staining and directional MBP nerve fibers. Co-cultures of Schwann cells/neurons were transduced with adenoviruses coding for GFP (A, B and C), QKI-6 (D, E and F), QKI-7 (G, H and I), or the combination of QKI-6 and QKI-7 (J, K and L). After infection, co-cultures were allowed to myelinate for 5 days with ascorbic acid. Cells were immunostained with anti-p27^KIP1^ antibodies (red), anti-MBP antibodies (purple) and visualized by fluorescence confocal microscopy. The green cells denote infected Schwann cells as well as infected axons. Ectopic expression of QKI-6 (E), QKI-7 (H) and QKI-6/7 combination (K) activate the expression of p27^KIP1^ in the nucleus of Schwann cells. Schwann cells are in the vicinity of MBP+ axons fibers (F, I and L). The inset (L) is an image showing a merge from p27^KIP1^-positive Schwann cells and MBP-positive axons (arrowheads). (M) This image shows the double staining for p27^KIP1^ (red) and MBP (purple) from a combined infection by QKI-6 and QKI-7 adenoviruses and demonstrate that p27^KIP1^ Schwann cells are associated with MBP-positive axonal internodes (arrows). (N) Denotes the green fluorescence of the GFP-positive cells of panel M. (O) Schwann/DRGN co-cultures infected with control or the indicated QKI adenoviruses were lysed and the proteins analyzed by immunoblotting for p27^KIP1^ and β-actin to ensure equal loading. The relative band intensities quantified by densitometry are indicated below.

The expression of p27^KIP1^ is required for the cell cycle arrest and differentiation of oligodendrocyte [33], [34], [35], [36] and Schwann cells [37], [38]. As the QKI-6 and QKI-7 isoforms have been shown to up-regulate p27^KIP1^ protein levels [8], we investigated whether the QKI isoforms regulate p27^KIP1^ in myelinating Schwann cells. The Schwann cell/DRGN cultures were transduced with QKI-6 and QKI-7 expressing adenoviruses and stained for p27^KIP1^ and MBP by indirect immunofluorescence. QKI-6, QKI-7 or the combination of QK-6/-7 induced an increase in the number of MBP-positive axonal segments (Figure 4, panels C, F, I, and L). These augmentations in myelination correlated with the increased number of p27^KIP1^ positive nuclei (panels B, E, H, and K) that was significantly higher in QKI-6/7 as compared to GFP-transduced co-cultures (Figure 4, compare panels B and K). Interestingly, the myelinating Schwann cells that were wrapped around axons stained positively for p27^KIP1^, demonstrating that nuclear p27^KIP1^ expression is a marker of myelinating Schwann cells. Confocal microscopy confirmed that p27^KIP1^ positive Schwann cells are in close contact with myelinated axons (Figure 4, panels M and N). We next examined the change in p27^KIP1^ protein levels by immunoblotting with anti- p27^KIP1^ antibodies. p27^KIP1^ proteins levels were increased ∼1.7-fold in QKI-6, QKI-7 and QKI-6/7 expressing co-cultures in comparison to control cultures over-expressing GFP (Figure 4, panel O). In addition, we examined the expression level of Krox-20 an early transcription factor that specifically regulates Schwann cell differentiation and found no significant change in QKI-6 and/or QKI-7 transduced cultures (data not shown). Together these findings indicate that that QKI-6 and QKI-7 promote Schwann cell maturation by inducing p27^KIP1^ dependent cell cycle arrest.

### QKI-deficient primary rat Schwann cells exhibit reduce MBP, p27^KIP1^ and Krox-20 mRNAs

**Figure 5:**
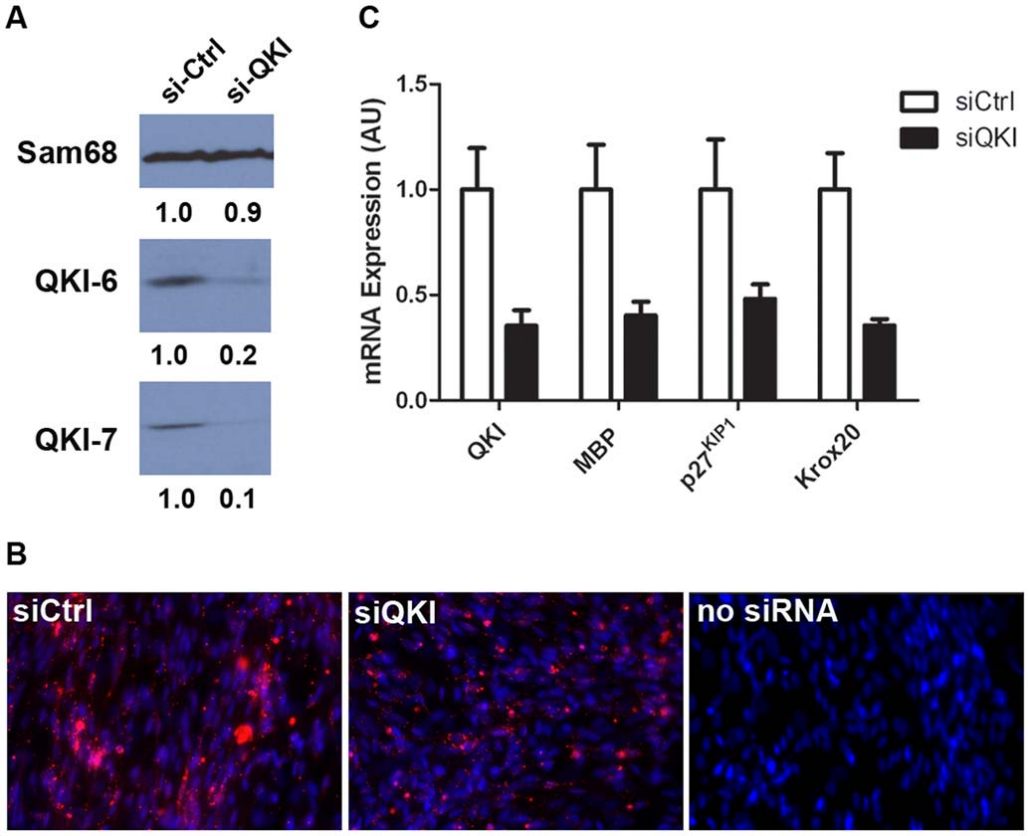
QKI-6 and QKI-7 deficient Schwann cells have reduced levels of MBP, Krox-20 and p27^KIP1^ mRNAs. (A) C6 rat glioma cells were transfected with QKI-specific siGENOME SMARTpool or luciferase specific siRNAs control (siCtrl), lysed and proteins were immunoblotted for QKI-6 and QKI-7 isoforms; Sam68 was used to ensure equal loading. The quantification by densitometric scanning is indicated below. (B) Co-cultures of Schwann cells/neurons were transfected with siCtrl or QKI-specific siGENOME SMARTpools in combination with siGlo, a transfection indicator. Transfection efficiency was assessed by the percentage of DAPI stained Schwann cells (blue) that incorporated siGlo (red) in the perinuclear region, and siGlo signal was absent in non-transfected co-cultures. (C) The mRNA levels of QKI, MBP, p27^KIP1^ and Krox-20 in siRNA-treated was quantified by real-time RT-PCR. The bars depict the mean plus the standard error of the mean (error bars) from three separate experiments.

To confirm the positive regulatory role of ectopically expressed QKI-6 and QKI-7 in Schwann cell maturation, we used a knockdown approach using RNA interference to reduce the expression of the endogenous QKI-6 and QKI-7 isoforms. We first verified the efficiency of siGENOME SMARTpool siRNA targeting the rat QKI isoforms in C6 rat glioma cells. C6 glioma cells were transfected with QKI siRNAa for 72 hrs and the prepared cell lysates were immunoblotted with anti-QKI-6 or anti-QKI-7 antibodies and anti-Sam68 antibodies were used as a loading control. We observed that in comparison to control cultures (siCtrl), both QKI-6 and QKI-7 isoforms were down-regulated by >80% by QKI-specific siRNA (siQKI, Figure 5A). We next reduced the levels of QKI expression in Schwann/DRGN cultures using the same strategy except we used a more sensitive assay, quantitative RT-PCR, to monitor knockdown in rat Schwann cells [39]. To monitor the efficiency of siRNA delivery in the Schwann/DRGN cultures, we also included the siGlo transfection indicator. We observed a >70% transfection efficiency (Figure. 5B) which correlated with an ∼50% reduction in QKI isoform expression, as assessed by qRT-PCR (Figure 5C). Interestingly, a significant reduction in MBP, p27^KIP1^, and Krox-20 mRNA levels normalized to GAPDH (glyceraldehyde-3-phosphate dehydrogenase) was observed (Figure 5C). Together these data further suggest that the QKI isoforms are essential regulators of Schwann cell myelination.

## Discussion

In this report we present evidence that the QKI proteins are determinants of Schwann cell differentiation. First we show that the expression of QKI-6 and QKI-7 positively regulates Schwann cell differentiation, as they promote cell cycle arrest by inducing p27^KIP1^ expression. This is further confirmed by the increased ability of QKI-6 and QKI-7-transduced Schwann cells to produce MBP and myelinate axons of DRGN in primary cultures. Furthermore, in QKI-deficient Schwann cells we observe down-regulation of the level of MBP mRNA as well as other Schwann cell maturation markers, p27^KIP1^ and Krox-20. Our findings show that both QKI-6 and QKI-7 potentiate Schwann cell myelination, and they exert synergistic effects when combined.

The role of RNA binding proteins or the post-transcriptional events regulating peripheral myelination is unknown [1]. We showed previously that QKI-6 and QKI-7 are positive regulators of oligodendrocyte differentiation and myelin production [8]. We now extend these finding showing a role in peripheral myelination by Schwann cells. The visualization of the myelin sheath by electron microscopy permitted a close examination of the myelin sheath structure. The myelin sheaths from the QKI-6 and QKI-7 transduced co-cultures were significantly thicker, but occasional aberrations such as cytoplasmic inclusions were observed. The reason for the appearance of the occasional aberrations is unknown, however, we suspect that this may be caused by continuous expression of the QKI-6 and QKI-7 isoforms in our expression system. These findings imply that the absence of QKI-6 and QKI-7 in the *qk^v^* mice may be the main cause of the myelination defects seen in the peripheral nerves of these mice.

The QKI proteins are phosphoproteins often referred to as STAR (signal transduction and activation of RNA) proteins [11], [12]. Indeed QKI is a known substrate of the soluble tyrosine kinase p59*^fyn^* and its phosphorylation negatively regulates its association with RNA [40]. These observations suggest that the balance between the nuclear and cytoplasmic QKI isoforms and/or the QKI RNA binding activity may be regulated by signaling pathways.

The peripheral myelination induced by the ectopic expression of QKI-6 and QKI-7 coincided with the elevated protein expression of p27^KIP1^and MBP. The differentiation of oligodendrocytes requires their withdrawal from the cell cycle, and the accumulation of p27^KIP1^ precedes oligodendrocyte differentiation [33], [36], [41]. Moreover, the migratory and differentiation capabilities of glial progenitors are perturbed during E13 to E19 in p27^KIP1^ null mice, as there were significant increases in QKI-positive; BrdU-positive cells in the subventricular zone compared to wild-type controls [42]. In addition, reduction in MBP staining is observed in p27^KIP1^ knockout mice in the optic nerve, consistent with the requirement for p27^KIP1^ and PNS myelination [43]. We have previously showed that p27^KIP1^ increases after the ectopic expression of QKI-6/7 in oligodendrocytes [8]. Moreover, the p27^KIP1^ mRNA is stabilized and a QRE is localized within the coding region of p27^KIP1^ and bound by QKI [13]. In the present manuscript, we show that the QKI-6/7 expression in Schwann cells results in increased myelination and nuclear p27^KIP1^ expression. On the contrary, repressing QKI expression using RNA interference resulted in suppression of its target mRNAs including p27^KIP1^, possibly by rendering the unbound p27^KIP1^ mRNA susceptible to degradation. These findings suggest that p27^KIP1^ is a positive marker of PNS myelination and that QKI is upstream of p27^KIP1^ in both the CNS and PNS.

In addition to p27^KIP1^, Krox-20 is another major positive regulator of Schwann cell maturation. The production of Krox-20 mRNA is known to occur within 1 hr of contact with an axon or after growth factor stimulation hence its alternative name early growth factor (Egr2) [44]. It has been demonstrated that peripheral nerves from Krox20-deficient mice are characterized by arrested myelination and decreased myelin protein levels [21], [22]. The post-transcriptional events regulating Krox-20 are unknown, but the Krox-20 mRNA harbors a perfect QRE that is bound by QKI *in vitro* [13]. In Krox-20 null mice, the immature Schwann cells are able to form a one-to-one relationship with axons. The defect underlies within the inability of Schwann cells to become arrested prior to differentiation, and their low levels of MBP expression [21], [45]. The *qk*-deficient mice die at E9.5 and have an open neural tube, thus neuronal and glial cell fate information could not be obtained from these animals [46]. The neural tube defect is similar as that exhibited by the Krox-20 null mice [47]. Furthermore, forced expression of Krox-20 in Schwann cells causes a block in Schwann cell proliferation [23], as observed with the QKI-6 and QKI-7 (Figure 1), suggesting that the QKI proteins might be linked to Krox-20 regulation or vice versa. Although the timing of QKI overexpression in myelinating Schwann/DRGN cocultures did not allow us to detect a significant Krox-20 induction, we observed a reduction in Krox-20 transcript in QKI-deficient Schwann cells. Thus it is tempting to speculate that QKI stabilizes Krox-20 mRNA similarly to p27^KIP1^, thus ensuring sufficient transcripts were accumulated in Schwann cells prior to the onset of myelination. If the relationship between Krox-20 and QKI is indeed correct, then human peripheral neuropathies may harbor mutations within the *qk* gene.

In conclusion, we have shown that the ectopic expression of QKI-6 and QKI-7 isoforms promotes Schwann cell maturation and myelination, as assessed by MBP expression. Interestingly the myelinating Schwann cells contained nuclear p27^KIP1^ suggesting that p27^KIP1^ is a marker of myelinating Schwann cells. Our findings show decreased levels of MBP, p27^KIP1^ as well as Krox-20 mRNAs in QKI-deficient Schwann cells. Our findings reveal that the QKI proteins are potent inducers of Schwann cell differentiation and strategies to up-regulate or over-express QKI-6 and QKI-7 isoforms may be useful to enhance peripheral nerve myelination.

## Materials and Methods

### Primary co-cultures of Schwann cells/neurons from rat dorsal root ganglion (DRG)

Schwann cells/neurons co-cultures were prepared using methods described previously [48]. DRGs were obtained from Sprague-Dawley rat embryos at 15–16 days gestation from Charles River Laboratories (St. Constant, QC, Canada). Embryos were collected in Leibovitz's (L-15) medium, the spinal column was dissected from each embryo and DRGs were plucked from the spinal cords and collected in fresh L-15 medium. DRGs were dissociated with trypsin (0.025 % in Hank's BSS) at 37°C for 15 min, followed by treatment with soybean trypsin inhibitor (5 mg/ml in L-15). The dissociated cells were resuspended for plating in defined medium consisting of DMEM/F12 containing N1 supplement, 0.09% BSA, 12 ng/ml 2.5 S NGF and antibiotics (penicillin/streptomycin) and cells from 72 DRGs were plated onto each rat tail collagen-coated 24-well plates. Cell cultures were maintained in serum-free N1 medium until their use after 21 days in culture. Myelination was initiated by adding fresh media containing 50 µg/ml of ascorbic acid. At the same time, co-cultures were infected with recombinant adenoviruses as previously described [6]. Five days after infections, cells were used for immunostaining experiments and/or immunoblot experiments. In each adenovirus treatment, 3 different wells were used by experiment. Antibodies used were for MBPs (Rabbit anti-MBP; 1∶500), p27^KIP1^ (Mouse anti-p27, 1∶1000, Chemicon) and for β−actin (Mouse anti-β-actin, 1∶500, Chemicon). Goat anti-mouse and goat anti-rabbit secondary antibodies conjugated to Alex546 (Molecular Probe) and Cy5 (Jackson Immunoresearch) were used for double-labeling experiments. Confocal analysis was carried out on a Zeiss microscope. The experimental protocol for preparation of primary cell cultures was approved by the McGill University Animal Care Committee and meets the guidelines of the Canadian Council on Animal Care.

### Adenovirus Construction and Infection

The co-cultures were infected with the indicated adenovirus co-expressing QKI-6 and QKI-7 driven by the TR5 (Tet-Off) promoter and GFP is under the control of the CMV promoter as described previously [4]. A multiplicity of infection (MOI) of 50 was sufficient to infect ∼90% of the cells, as judged by GFP positive cells.

### Electron Microscopy

Adenoviruses treated co-culture were rinsed in PBS, fixed in 2.5% glutaraldehyde in 15mM sodium cacodylate (pH 7.4) for 60 min, and postfixed for 1 hr in 1% osmium tetroxide and embedded for EM. Ultrathin sections were cut with a diamond knife and stained with uranyl acetate and examined by transmission electron microscopy (McGill University, Anatomy and Cell biology department). A myelin layers analysis was conducted on a population of 30 axons by treatment from 3 replicates experimental sets. The g-ratio was calculated by dividing the axonal diameter by the total fiber diameter. The statistical significance of myelin lamellae for each QKI adenoviruses treatment versus the control AdGFP was determined by two-sample t test ANOVA for assumption test and Tukey-Kramer Multiple Comparisons Test for P value of 0.001. InStat3 software (GraphPad Inc.) was used for graph and statistic analysis. (Define G ratio used and its meaning).

### Bromodeoxyuridine incorporation and cell cycle analysis

For analysis of BrdU incorporation using immunofluorescence staining, coculture containing Schwann cells and DRG neurons cells were infected for 24 h with adenoviruses (m.o.i: 50) expressing the different QK isoforms or GFP control. Culture progenitors were given a 16 h pulse with 10 µM of 5-bromo-2′-deoxyuridine (BrdU) and then fixed in 4% paraformaldehyde. Cells were permeabilized with ice cold acetone/methanol (1∶1). After 30 min in 1N HCl and neutralization with sodium borate buffer, cells were incubated with monoclonal anti-BrdU antibody (1∶100, Chemicon International) in PBS + 0.1%Triton + 5% goat serum for 3 hours. A Rabbit anti-mouse Alexa 546 (Molecular Probes) antibody diluted 1∶1000 in PBS; 0.1% Triton was used for detection.

### RNAi and Transfections

QKI-specific siGENOME SMARTpool siRNA purchased from Dharmacon (M115062-00-0010), and luciferase specific control siRNA (CGUACGCGGAAUACUUGA) were prepared according to manufacturer's instructions. 100nM of each siRNA plus 50nM of siGlo indicator (Dharmacon) were delivered to each well of a 24-well plate containing day-21 Schwann cell/DRGN co-cultures using Lipofectamine RNAiMAX. The transfection was repeated once every two days for three times, and cells were harvested on day-28 of culture. Monolayer of C6 rat glioma cells were transfected for 72 hours using conditions above.

### Quantitative real-time PCR

Total RNA was harvested using the RNeasy Plus Minikit (Qiagen) and reverse transcribed with Super Script II Reverse Transcriptase (Invitrogen). Intron-spanning pre-designed primers purchased from Qiagen were used at concentrations specified by the manufacturer. Quantitative PCR was performed using Quantitect SYBR Green qPCR kit (Qiagen) on Applied Biosystem 7500 real-time PCR system. The relative expression values for each gene of interest normalized to glyceraldehyde-3-phosphate dehydrogenase (GAPDH) were analyzed by ABI SDS software using the comparative CT method.

## Supporting Information

Figure S1 QKI encoding adenovirus effectively produces QKI-6 and QKI-7 in Schwann cells (A) Primary Schwann cell/neuron co-cultures were infected for 4 days with control GFP adenovirus (Adv GFP), or the combination adenoviruses encoding myc-tagged QKI-6 or QKI-7 (Adv QKI-6/7). Cultures were lysed, and proteins were immunoblotted for QKI-6 and QKI-7 to reveal the endogenous and the recombinant respective QKI isoforms. Both a short exposure of 10 sec and a longer exposure 10 min is shown. The myc-epitope tag was used to evaluate the relative levels of the each recombinant QKI isoform in infected cultures and Sam68 was used to ensure equal loading. The molecular mass markers are shown on the left in kDa and migration of the myc-epitope tagged QKI (myc-QKI) and the endogenous QKI (QKI) isoforms is shown. (B) The over-expression of recombinant QKI isoforms was quantified by densitometric analysis compared to the endogenous levels in arbitrary units (AU).
(2.48 MB TIF)

Figure S2 Expression of the QKI isoforms in the mouse brains during development. (A) Mouse brains were harvested on embryonic day 17.5, 18.5 (E17.5, E18.5) and postnatal day 1, 3, 6, 8, 10, 15, 30 (P1–P30), homogenized and lysed. Protein lysates were immunoblotted for QKI-5, QKI-6 and QKI-7 as indicated. β-actin was used to ensure equal loading. (B) The expression levels of each QKI isoform normalized to loading control was quantified by densitometry analysis.
(5.48 MB TIF)
