# Supplementary figures and images for: The QKI-6 and QKI-7 RNA Binding Proteins Block Proliferation and Promote Schwann Cell Myelination
Source: PLoS One. 2009 Jun 11;4(6):e5867. doi: 10.1371/journal.pone.0005867 (PMC2690695; doi:10.1371/journal.pone.0005867)

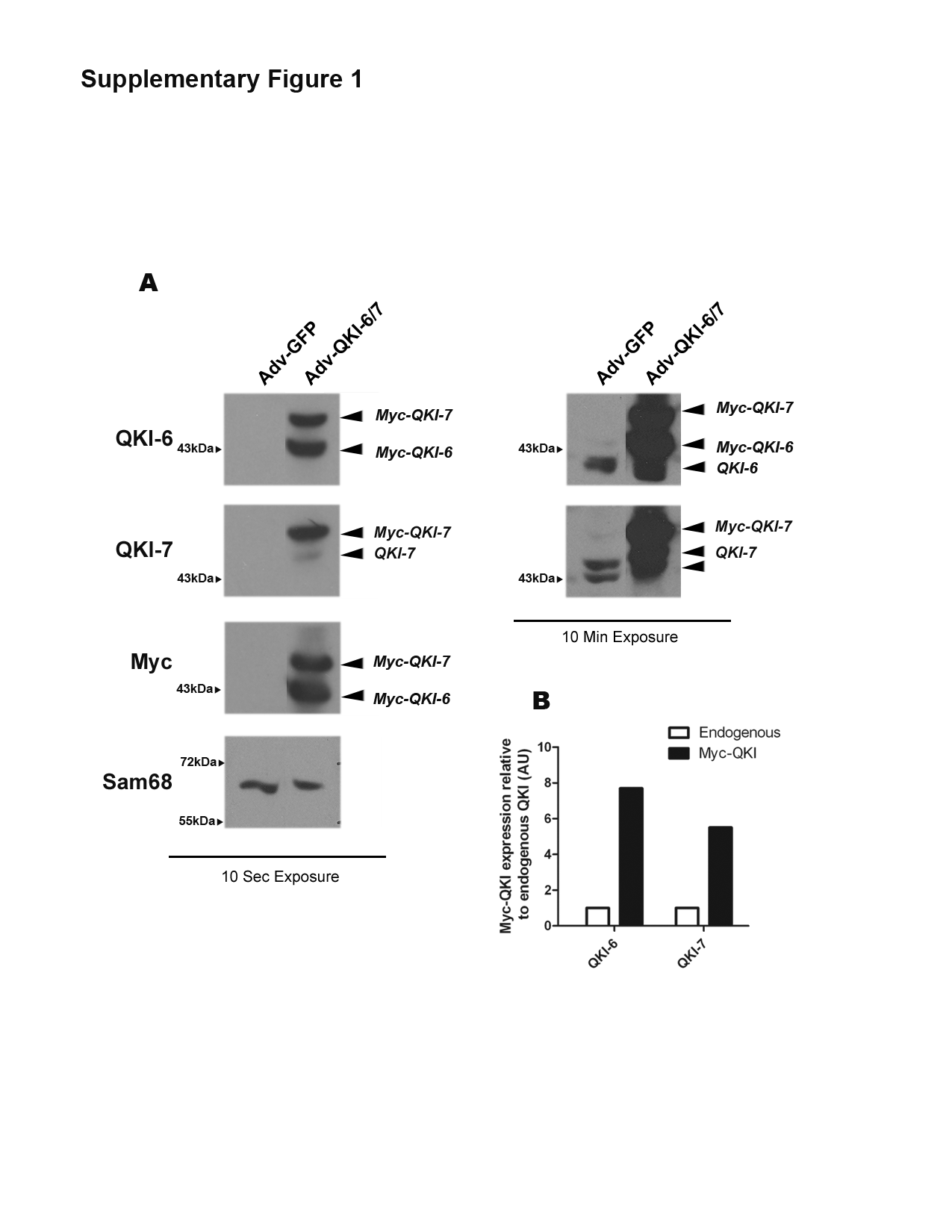

Supplement: Figure S1 — QKI encoding adenovirus effectively produces QKI-6 and QKI-7 in Schwann cells (A) Primary Schwann cell/neuron co-cultures were infected for 4 days with control GFP adenovirus (Adv GFP), or the combination adenoviruses encoding myc-tagged QKI-6 or QKI-7 (Adv QKI-6/7). Cultures were lysed, and proteins were immunoblotted for QKI-6 and QKI-7 to reveal the endogenous and the recombinant respective QKI isoforms. Both a short exposure of 10 sec and a longer exposure 10 min is shown. The myc-epitope tag was used to evaluate the relative levels of the each recombinant QKI isoform in infected cultures and Sam68 was used to ensure equal loading. The molecular mass markers are shown on the left in kDa and migration of the myc-epitope tagged QKI (myc-QKI) and the endogenous QKI (QKI) isoforms is shown. (B) The over-expression of recombinant QKI isoforms was quantified by densitometric analysis compared to the endogenous levels in arbitrary units (AU). (2.48 MB TIF) [file pone.0005867.s001.tif]

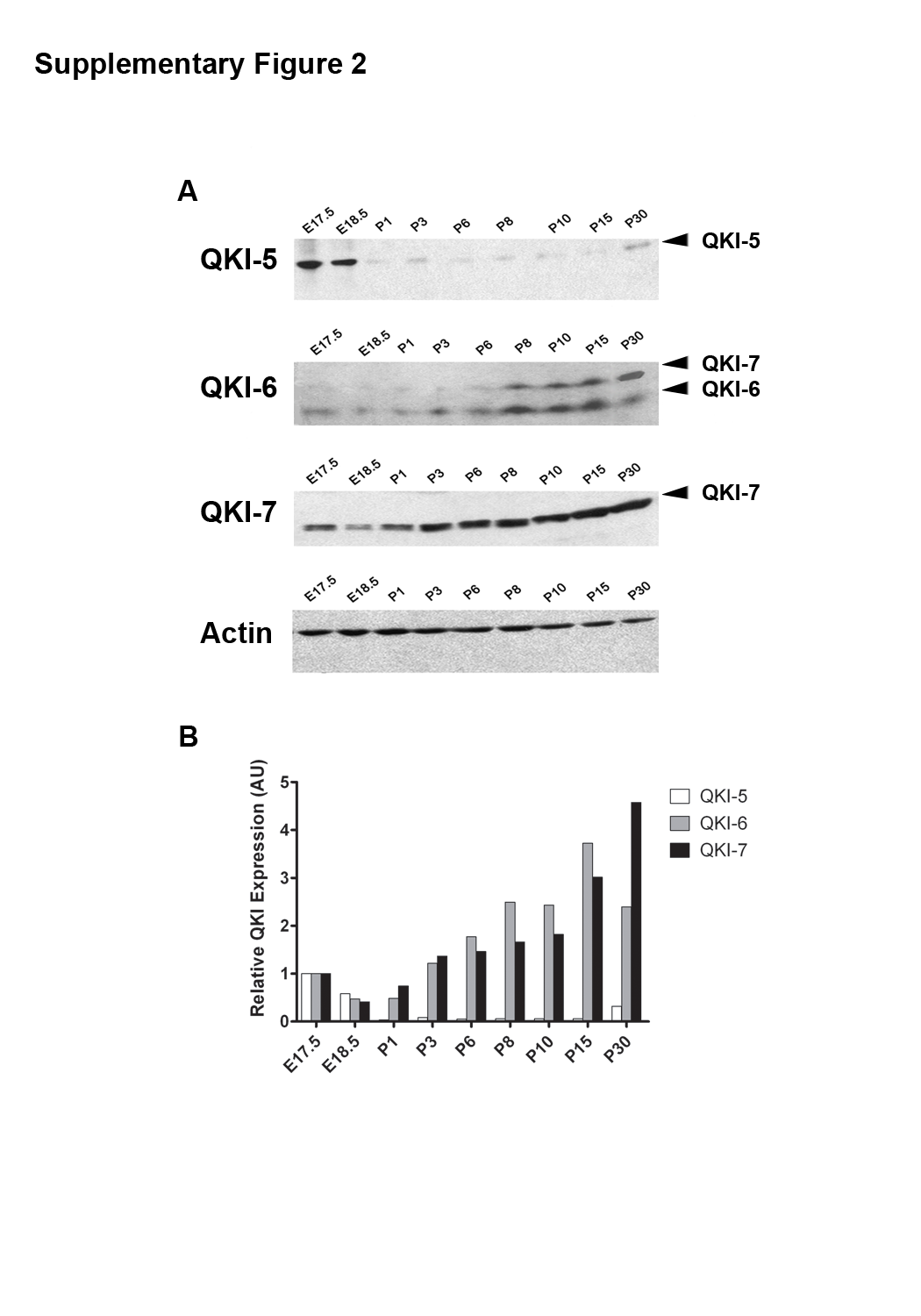

Supplement: Figure S2 — Expression of the QKI isoforms in the mouse brains during development. (A) Mouse brains were harvested on embryonic day 17.5, 18.5 (E17.5, E18.5) and postnatal day 1, 3, 6, 8, 10, 15, 30 (P1–P30), homogenized and lysed. Protein lysates were immunoblotted for QKI-5, QKI-6 and QKI-7 as indicated. β-actin was used to ensure equal loading. (B) The expression levels of each QKI isoform normalized to loading control was quantified by densitometry analysis. (5.48 MB TIF) [file pone.0005867.s002.tif]
